# Automatic Pose Recognition for Monitoring Dangerous Situations in Ambient-Assisted Living

**DOI:** 10.3389/fbioe.2020.00415

**Published:** 2020-05-14

**Authors:** Bruna Maria Vittoria Guerra, Stefano Ramat, Giorgio Beltrami, Micaela Schmid

**Affiliations:** Laboratory of Bioengineering, Department of Electrical, Computer and Biomedical Engineering, University of Pavia, Pavia, Italy

**Keywords:** Ambient-Assisted Living, vision-based activity recognition, skeleton tracking, pose recognition, machine learning, geometric features

## Abstract

Continuous monitoring of frail individuals for detecting dangerous situations during their daily living at home can be a powerful tool toward their inclusion in the society by allowing living independently while safely. To this goal we developed a pose recognition system tailored to disabled students living in college dorms and based on skeleton tracking through four Kinect One devices independently recording the inhabitant with different viewpoints, while preserving the individual’s privacy. The system is intended to classify each data frame and provide the classification result to a further decision-making algorithm, which may trigger an alarm based on the classified pose and the location of the subject with respect to the furniture in the room. An extensive dataset was recorded on 12 individuals moving in a mockup room and undertaking four poses to be recognized: standing, sitting, lying down, and “dangerous sitting.” The latter consists of the subject slumped in a chair with his/her head lying forward or backward as if unconscious. Each skeleton frame was labeled and represented using 10 discriminative features: three skeletal joint vertical coordinates and seven relative and absolute angles describing articular joint positions and body segment orientation. In order to classify the pose of the subject in each skeleton frame we built a two hidden layers multi-layer perceptron neural network with a “SoftMax” output layer, which we trained on the data from 10 of the 12 subjects (495,728 frames), with the data from the two remaining subjects representing the test set (106,802 frames). The system achieved very promising results, with an average accuracy of 83.9% (ranging 82.7 and 94.3% in each of the four classes). Our work proves the usefulness of human pose recognition based on machine learning in the field of safety monitoring in assisted living conditions.

## Introduction

The integration of *frail* people into society is a major issue in developed countries for both social and economic motivations. This inclusion should start with the environment in which these subjects live, and can be achieved by improving well-being, autonomy, care, and assistance in the home. Internet of things (IoT) and modern domotic technologies offer a plethora of solutions to implement intelligent and automated environments allowing *frail* individuals to live in autonomy and safety in place (Álvarez-García, 2013; Amiribesheli et al., 2015; Lloret et al., 2015; Debes et al., 2016; Mehr et al., 2016; Majumder et al., 2017; Guo et al., 2019). In the last years, Ambient-Assisted Living (AAL) has attracted great attention and numerous projects have proposed different networks of sensors and complex monitoring algorithms which most frequently require to shift from a low-level data collection and analysis toward high-level information integration, context processing, activity recognition and inference (Chen et al., 2012; Verrini et al., 2018).

The most commonly used sensors for AAL are wearable and environmental sensors (Delahoz and Labrador, 2014; Pannurat et al., 2014; Mehr et al., 2016; Torti et al., 2019). The first category includes radio frequency identification tags, accelerometers, gyroscopes, and more generally inertial sensors which can be embedded in devices such as smartphones and smartwatches. The main advantages of wearable sensors are to be particularly light and non-intrusive, yet they have the important drawback of being dependent on rechargeable batteries and of requiring correct body positioning to maximize the signal quality and reduce noise.

The second category, environmental sensors, commonly refers to cameras able to monitor an inhabitant behavior and environment changes (vision-based activity recognition) (Chen et al., 2012). Using properly located cameras, the inhabitant can be recorded while free to perform the normal actions of daily life without limitations and without having to be in anyway involved, e.g., having to remember to wear a device or to charge it. The cameras used for AAL purposes are commonly depth cameras, such as Asus Xtion (Taipei, Taiwan), Intel RealSense (Santa Clara, United States), Orbbec Astra (Troy, United States) and Microsoft Kinect (Redmond, United States) (Ben Hadj Mohamed et al., 2013; Han et al., 2013; Gasparrini et al., 2014; Mastorakis and Makris, 2014; Pannurat et al., 2014; Visutarrom et al., 2014, 2015; Damaševičius et al., 2016; Calin and Coroiu, 2018). Thanks to many approaches based on RGB sequences, depth images or their combination, these sensors are able to provide detailed information about 3D human motion (Wang et al., 2014; Kim et al., 2017). Moreover, real time algorithms can estimate the body skeleton, which allows to describe human poses with a lower dimensionality than RGB/RGB-D-based representations while being intrinsically anonymous, thereby respecting the privacy of the subject.

To infer what an individual is doing, and which pose he/she assumes, the data collected from both wearable sensors and cameras are commonly processed using data mining, machine learning (ML), and deep learning (DL) algorithms. Machine learning focuses on teaching computers how to learn from experience, without the need to be programed for specific tasks. This makes ML particularly suitable to analyze data coming from smart house sensors in order to recognize falls or to detect a dangerous situation during daily life activities. Machine learning algorithms such as Naïve Bayes classifiers (NBC), K-nearest neighbor (KNN), support vector machines (SVM), hidden Markov models (HMM), and artificial neural networks (ANN), random forest (RF), decision tree (DT), and logistic regression (LR) (Begg and Hassan, 2006; Crandall and Cook, 2010; Hussein et al., 2014; Visutarrom et al., 2014; Wang et al., 2014; Amiribesheli et al., 2015; Jalal et al., 2015) are the most popular algorithms used in sensor- and vision-based activity recognition. K-nearest neighbor is widely used in real-life scenarios since it is non-parametric, meaning that it does not make any assumptions about the underlying distribution of the data. The main disadvantage of this approach is that the algorithm must compute the distance and sort all the training data at each prediction, therefore it turns out slow with large numbers of training examples. A similar weakness affects the SVM algorithm, which nevertheless is considered relatively memory efficient. Achieving the best classification results, for any given problem, requires setting several key parameters that need to be chosen correctly (Bishop, 2006). Artificial neural networks, such as multi-layer perceptron (MLP) algorithm, can be applied to many smart home problems, ranging from activity classification, to novelty and anomaly recognition, to activity prediction (Begg and Hassan, 2006; Hussein et al., 2014). Patsadu et al. (2012) compared four ML algorithms (MLP, SVM, DT, and NBC) training the models on a dataset of 7200 frames and testing them on further 3600 frames to identify three different human poses: standing, sitting, and lying down. The poses were performed by a subject positioned in front of the camera and each videoframe was encoded as a row of 20 body-joints positions that were used as features for ML algorithms. The best classifier was found to be the MLP network (100% of accuracy vs. 99.75% of SVM, 93.19% of DT and 81.94% of NBC). Visutarrom et al. (2014) went deeper into this topic comparing six different ML classifiers and two different sets of features (geometric vs. skeletal joints features). Four poses (standing, sitting, sitting on floor, and lying down) of a subject watching television in front of the Kinect device were classified. They compared MLP, DT, NBC, RF, LR, and SVM by training and testing the six models on a dataset of geometric features and found that DT, RF, and MLP algorithms performed best (accuracy about 97.9%), followed by the SVM (accuracy about 97.5%). Altogether various ML algorithms have been successfully applied to pose recognition, yet all these approaches suffer from various limitations that do not prove their usefulness in the context of identifying dangerous situations in ecological conditions of assisted living. Indeed, all algorithms were applied to recordings performed by subjects statically facing the camera, i.e., the ideal conditions for skeleton tracking systems, which are nonetheless unlikely to occur while monitored subjects perform their daily living activities at home. Furthermore, their performance has been tested in recognizing upright standing or poses typically assumed immediately after a fall, e.g., lying down or sitting on the floor, yet omitting more general dangerous situations such as recognizing that a person has fainted while sitting on a chair.

In an automated monitoring system for AAL, the accuracy of event recognition is vital. False negatives are unacceptable as they would imply the lack of intervention in a dangerous condition. Recognition accuracy is strongly dependent not only on the model algorithm, but also on the type and number of attributes that make up the database used to train the network. In vision-based action recognition, the common approach is to extract image features from video data and to issue a corresponding action class label (Poppe, 2010; Babiker et al., 2018). Nevertheless, when skeleton representation of the human body is used, the most privileged discriminative features are the raw data coming from the skeletal tracking (joint spatial coordinates) (Patsadu et al., 2012; Youness and Abdelhak, 2016) or some indices expressing geometric relations between certain body points, such as: the vertical distance from hip joint to room floor (Visutarrom et al., 2014, 2015), the distance between the right toe and the plane spanned by the left ankle, the left hip and the foot for a fixed pose (Müller et al., 2005) the distance between two joints, two body segments, or a joint and a body segment (Yang and Tian, 2014), the relative angle between two segments within the body kinematic chain (Müller et al., 2005) and finally, the size of the 3D bounding box enclosing the body skeleton (Bevilacqua et al., 2014). Geometric features are synthetic in the sense that they express a single geometric aspect making them particularly robust to spatial variations that are not correlated with the aspect of interest (Müller et al., 2005). In order to identify the best attribute set to classify, off- and on-line, standing, bending, lying, and sitting poses, Le et al. (2013) compared the results of a ML algorithm trained and tested with four different sets of features. They trained an SVM with a radial basis function kernel on off-line data referred to a subject in front of the camera, using 7, 9, and 17 joint angles with and without scaling, and absolute joint coordinates without scaling. In off-line, optimal Kinect acquisition configuration, very good results were obtained with the absolute coordinates without scaling. They then tested the algorithm also using on-line data of a subject at a different distance from and at different angles with the Kinect camera. In the latter, more realistic scenarios, the angles were found to represent more relevant features for posture representation.

In this paper we focus on the problem of skeleton-based human pose recognition for the detection of dangerous situations. This work is part of a broader project (TheDALUS, The Disable Assisting Living for University Students), aimed to promote the inclusion of disabled students in a university environment (a room in college dorms) guaranteeing them safety and autonomy. This is done using a net of four Kinect One devices, whose data are collated and processed to identify both voluntary requests for help and dangerous situations (i.e., the subject has fainted or slipped from the wheeling chair, etc.) to trigger an alarm toward third parties, when needed. During daily activities a subject assumes a set of poses that can be very similar to those assumed during dangerous situations. Our approach is based upon the consideration that to distinguish these two different scenarios knowledge of the location domain is fundamental (the spatial position of the room inhabitant, objects and room furniture position and the relative position of the inhabitant with respect to the objects and the room furniture). Indeed, a normal pose could become a dangerous one when it takes place in relatively specific locations of the room. For example, the lying down pose is a daily living pose if it occurs on the bed. Conversely, it takes the form of a possible alarm condition if it occurs on the floor. In this context, an accurate body pose pattern recognition model must be defined first, and, in a later processing stage, the identified poses can be joined with the knowledge of the location domain. This implicit relationship between body poses and related spatial context provides the heuristics to infer the occurrence of a dangerous scenario, thereby broadening the scope of current approaches of ML in human pose recognition to the field of monitoring safety in assisted living conditions. The aim of this study is to implement the first step of this analysis procedure by using a large amount of skeleton tracking data referred to real scenarios, in which a more extensive camera coverage of the room is obtained by using four Kinect One devices. As such, here we are interested in classifying each acquired skeleton frame provided by the device in a set of predefined poses (standing, sitting, lying down and “dangerous sitting”). To this goal a three layers MLP network was trained and tested using a custom-built data set of robust and discriminative kinematic features computed based on skeleton data.

## Materials and Methods

### Experimental Set-Up

In order to minimize the invasiveness of the monitoring system, a main requirement in a 24-h surveillance of daily activities setting, we decided to avoid any wearable sensor. On the other hand, considering the constraints raised by the privacy of the students inhabiting the rooms, video recording and video surveillance systems did not represent a viable option. We therefore chose a motion sensing system based on skeletal tracking (Booranrom et al., 2014; Du et al., 2015; Gasparrini et al., 2015; Visutarrom et al., 2015; Liu et al., 2018). The current implementation is based on Microsoft’s Kinect One motion sensing system, yet it is easily portable to any skeletal tracking device that can provide the 3D coordinates of the chosen set of skeletal joints. The Kinect One motion sensing system can detect a human body and voice signal using an RGB camera (1920 × 1080 pixels), a depth sensor (512 × 424 pixels) and an array of four microphones (48 kHz). The depth sensor is composed of an IR emitter and an IR camera and provides depth measurements based on the Time of Flight principle (Pagliari and Pinto, 2015; Sarbolandi et al., 2015; Corti et al., 2016). Acquisitions can be carried out with a framerate up to 30 Hz and require a computer with an USB 3.0 interface for data transfer. The ideal distance of an object from the sensor is 0.8–3.5 m, with a maximum range of 0.5–4.5 m. The angle of vision is 60° vertically and 70° horizontally (Sell and O’Connor, 2014; Fankhauser et al., 2015; Pagliari and Pinto, 2015). Microsoft released also a Software Development Kit (SDK), used for skeletal estimation. It is capable of tracking 25 joints for up to six users simultaneously (Microsoft, 2019).

**FIGURE 1:**
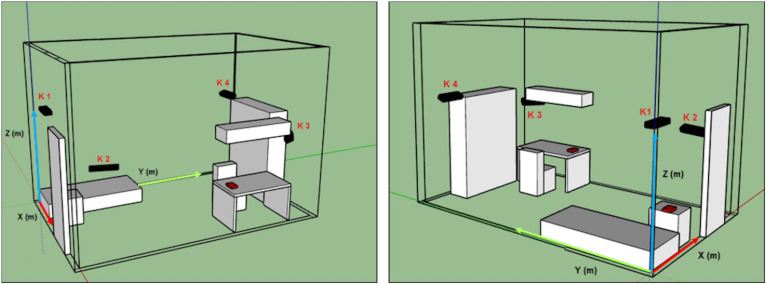
Kinect one positions in the prototype room (K1, K2, K3, and K4), reconstructed with a CAD software (SketchUp). Two different fields of view.

Experimental acquisitions were performed in a prototype room, mimicking that of the university college dorms (same dimensions and similar furniture) that was set up in the laboratory. In this setting, we decided to record each experimental trial using four Kinect One devices (K1, K2, K3, and K4 in Figure 1). Two are positioned to sense the whole room (K1 and K4), while the remaining two are placed to specifically acquire two areas of the room, such as the bed (K2) and the desk (K3), which were especially relevant to our aim. This decision was made after several careful eye-inspections of the different shots obtained with different camera configurations. Each arrangement was different for number, position and orientation of the devices. The goal was to ensure recording of the entire room minimizing possible blind spots. The data of the four Kinect One were acquired at the same time but processed separately. A custom-made C#-based tool with GUI was developed using VisualStudio 2017 to control the Kinect One acquisitions.

### Acquisitions

We decided to focus our acquisitions around the three most frequent and recurrent poses assumed by a person in a room during daily activities (Datasets – Advise, 2019; Fall detection Dataset, 2019; Fall detection testing dataset, 2019; Weblet Importer, 2019): standing, sitting, and lying down. In addition to the listed poses, we added one further pose, labeled “dangerous sitting,” which grouped all situations of malaise or fainting resulting in a seated person slumped or lying backward. This allowed us to perform a first distinction, prior to establishing a relationship between the subject location and the room furniture, between routine activities and alarm situations. Experimental protocols were designed to simulate the actions and poses performed during the daily life of a general disabled student, not necessarily having motor disabilities.

In order to build a dataset suitable for training a neural network to discern the four poses we performed a set of experimental acquisitions on a group of 12 normal subjects (7 females and 5 males; age ranging 25 and 60 years old; height ranging 1.55 and 1.90 m). All subjects gave written informed consent in accordance with the Declaration of Helsinki. The four Kinect One devices installed in the room acquired simultaneously the movement of the subject. The acquisitions were structured as four separate sessions performed on the same day for a total of about 13 min:

• subject starts to walk from standing position in front of K1 (Figure 1), then grabs a chair near the desk, placing it in front of the camera, and finally sits on it. While sitting, the subject first moves the head backward and then leans the trunk forward, while simultaneously pitching the head as an unconscious person. The subject then returns to the normal sitting position and finally gets up and brings the chair back to its original location. Each pose was maintained for 10 s. The sequence was then repeated in front of the other cameras (K2, K3, K4 in Figure 1);

• subject starts sitting on the bed, then lies down on the back, turns on the right side, then returns on the back and turns to the other side;
• subject starts lying on the ground on the back, then turns on the left side;
• subject starts sitting on the bed, then lies down. The action is repeated three times.

The sequence of poses in each acquisition was timed by the operator running the acquisitions.

### Data Pre-processing

**FIGURE 2:**
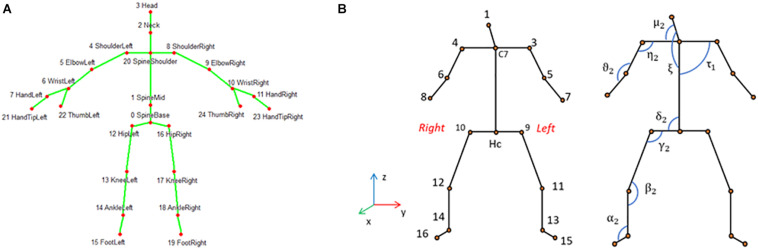
**(A)** 25-joints skeleton computed by Kinect One; **(B)** reduced skeleton used in this analysis with 17 joints for the left and right hemi-body. The relative angles computed in the plane defined by the two body segments are depicted, for illustrative purpose, only for the frontal plane and labeled with Greek letters. For clarity, except for τ_1_, only the angles of the right hemi-body are shown.

Using custom developed software based on the Kinect’s SDK we computed the spatial coordinates (*x*, *y*, *z*) of the standardized 25 skeletal joints (Microsoft, 2019). Based on considerations relative to the reliability of the detected joints and to the aim of this study, we decided to reduce the number of skeletal joints from 25 (Figure 2A) to 16. An additional joint labeled Hc was instead added as the midpoint between the two hips joints (Figure 2B).

The 17 selected joints were (Figure 2B): head (1), shoulders segment’s mid-point (C7), acromion (3–4), elbow (5–6), wrist (7–8), iliac crest (9–10), knee (11–12), malleolus (13–14), foot (15–16); hips segment’s mid-point (Hc). In order to identify the position of the subject in the room, the coordinates of the 17 joints were roto-translated to obtain data referred to an absolute reference system (*X*, *Y*, *Z*) located in one corner of the mock-up room (Figure 1). The absolute position in space of each body joint, described by the corresponding *X*, *Y*, *Z* triplet, isn’t the most convenient description for classifying human poses, since: (1) coordinates depend on the relative location of the individual in the room, while the same posture can be taken in different locations within the room; (2) the joint coordinates of two subjects having the same pose in the same room location have different values depending on the size of the subject’s body; and (3) posture is independent of where it occurs in space while it is defined by the geometrical relationship between the different body segments. The latter can instead be efficiently captured by articular angles, so that we chose to compute the following 16 articular angles defined between two consecutive body segments measured in the plane defined by the segments themselves: head–shoulder axis (μ_1_, μ_2_), head–trunk (ξ), shoulder axis–trunk (τ_1_, τ_2_), shoulder axis–arm (η_1_, η_2_), arm–forearm (θ_1_, θ_2_), trunk–iliac crest axis (δ_1_, δ_2_), iliac crest axis–thigh (γ_1_, γ_2_), thigh–leg (β_1_, β_2_), and leg–foot (α_1_, α_2_) (Figure 2B). Based on the same line of reasoning we further computed the roll and pitch angles of the head and trunk and labeled them as follows: A_pitch (trunk pitch), A_roll (trunk roll), B_pitch (head pitch), and B_roll (head roll). All angles were normalized dividing them by 180°. We further considered the vertical coordinates (*Z*) of the skeletal joints as they are significant for distinguishing the lying down from the standing pose. On the other hand these are not so discriminative for discerning between sitting and “dangerous sitting” poses, which are more easily identified through joints angles’ values. The joints’ *Z* coordinates werethen scaled on the height of each subject.

During the acquisition process we noted that sometimes Kinect One was not able to recognize the subject. For example, transient exits of the subject from the camera sight (Figure 1) could cause temporary non-identifications of all skeletal joints, and the same may occur when the subject assumes a “dangerous sitting” pose while not facing the camera. This could generate temporal holes between data frames (missing data). For these frames we decided to assign the value “999” to all the selected parameters in order to maintain consistency among the data of the four Kinect One systems. All the pre-processing algorithms were implemented using MATLAB.

### Database

Once all joints’ *Z*-coordinates, the relative angles and the chosen pitch and roll angles were obtained, i.e., a total of 37 (17 vertical joints coordinates, 16 relative angles, 4 absolute angles) features describing the skeleton in each frame. We then applied a ReliefF (Urbanowicz et al., 2018) algorithm for feature selection (MATLAB) and selected a subset of ten attributes: A_pitch, A_roll, B_pitch, B_roll, ξ, μ_2_, δ_2_, Z_1, Z_C7, Z_Hc (see Figure 2B for the last six attributes).

Using a custom-made LabView (National Instruments, Inc.) software, angles and joints position traces were then visually inspected together with a graphical visualization of the reconstructed skeleton to label each frame with one of the following four poses:

• Class 1: standing pose;
• Class 2: sitting pose;
• Class 3: lying down pose;
• Class 4: “dangerous sitting” pose.

Using the same software we also identified the frames corresponding to the transition from a pose to another and removed them from the dataset. The data from the four Kinect One systems were collated to build the final database composed by 602,530 frames. Among these, 145,196 frames belonged to Class 1, 233,593 to Class 2, 86,786 to Class 3, and 136,955 to Class 4.

A training set was eventually built using the data from 10 of the 12 subjects (database of 495,728 frames). The test set was built using the data of the 2 remaining subjects (database of 106,802 frames).

### Neural Network

**FIGURE 3:**
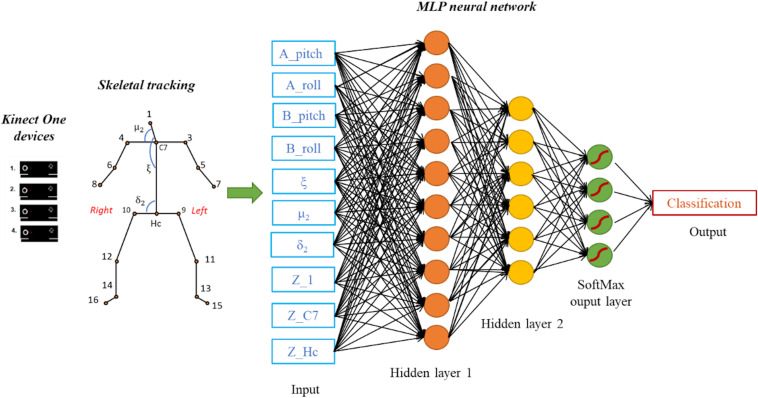
Data acquisition and processing with the proposed MLP network model having two hidden layers and a “SoftMax” output layer. The considered features are 10 kinematic parameters computed from skeletal tracking. The output classification corresponds to the one of the four classes (Class 1, Class 2, Class 3, Class 4).

The aim of this work was not to detect dynamic situations, such as the falling of the subject in order to prevent it, but rather to identify the subject lying on the floor immediately after the fall in order to activate an alarm and intervene with first aid actions. Therefore, in the current implementation we wanted to identify a subject pose at any one time, leaving the decision-making process about alarm triggering to a downstream algorithm having access to more data (e.g., subject’s position in the room). The pose classification problem is therefore seen as a static mapping problem. For this reason, among a range of possible ML algorithms, we have selected an MLP Neural Network to classify predefined human poses. The network was implemented in MATLAB using the Neural Network Toolbox. We designed a network consisting of three fully connected layers of neurons, plus an input layer connected to the 10 features describing each frame in the database (Figure 3). The first hidden layer has a number of neurons equal to the number of attributes in the database (10), each with a hyperbolic tangent transfer function and a bias. The second hidden layer has a structure similar to the first one, but contains a smaller number of neural units (6). The output layer is instead composed by a number of neurons equal to the number of target classes (4) and their transfer function is the “SoftMax” function producing, for each input element, the probabilities of belonging to each considered class. The MLP network was trained using the Levenberg-Marquardt backpropagation algorithm, first with a k-fold cross validation (*k* = 10), and then using the whole training set. The learning process was performed over a maximum of 1000 epochs, i.e., 1000 iterations on the training set.

### Statistical Analysis

MLP network was trained and tested 50 times to study its classification robustness. Total accuracy (mean over the four classes), class accuracy, F-score, sensitivity, and specificity were calculated for each network simulation. These parameters rely upon the concept of True Positive (TP, a pose correctly classified as pertaining to the considered class), True Negative (TN, a pose which is correctly classified as pertaining to a class different from the one considered), False Positive (FP, a pose that is wrongly classified as pertaining to the considered class), and False Negative (FN a pose that is wrongly classified as not pertaining to the class considered).

#### Accuracy

Accuracy is a metric parameter for evaluating classification models. In general, for binary classification, accuracy can be calculated as:

$$\text{Accuracy}=\frac{\text{TP}+\text{TN}}{\text{TP}+\text{TN}+\text{FP}+\text{FN}}$$

#### Sensitivity

The Sensitivity (also called Recall) is a metric parameter that measures the proportion of genuinely positive samples that are currently identified as such. It is defined as:

$$\text{Sensitivity}=\frac{\text{TP}}{\text{TP}+\text{FN}}$$

#### Specificity

The Specificity is the proportion of genuinely negative samples that are currently identified as such. It is defined as:

$$\text{Specificity}=\frac{\text{TN}}{\text{TN}+\text{FP}}$$

#### F-Score

F-score is an overall measure model’s accuracy that combines precision and recall. Precision is the number of positive results divided by the number of all positive results returned by a classifier. Recall, instead, is the ratio between TP and the number of all samples that should have been identified as positive, which corresponds to the sensitivity parameter.

$$\text{F-score}=2\times \frac{\text{Precision}\times \text{Recall}}{\text{Precision}+\text{Recall}}$$

where:

$$\text{Precision}=\frac{\text{TP}}{\text{TP}+\text{FP}}$$

For each of five parameters considered, the mean value over the 50 network simulations was then computed. This average operation was done only after verifying that the results listed above were normally distributed. Since the number of samples was 50, i.e., the number of network simulations, we decided to use the Shapiro–Wilk test as a hypothesis test (Hanusz and Tarasińska, 2015). The null hypothesis of this test is that the population is normally distributed. For each test performed the p-value was greater than the chosen alpha level, therefore the null hypothesis that the data came from a normally distributed population cannot be rejected (IBM SPSS Statistics, IBM). Therefore, in the result section, for each of the five parameters, the mean and the standard deviation are considered.

#### Confusion Matrix

Confusion matrix is a specific table summarizing the results of the classifier used to visualize the performance of a machine learning algorithm. The rows of the matrix represent the classifications predicted by the MLP network while the columns represent the instances actually belonging to each class.

In the present study we computed a confusion matrix for each of the 50 network simulations. Then, we computed a mean confusion matrix in which the number of frames reported in each cell is the mean, over the 50 confusion matrices, of the frames pertaining to that cell.

#### ROC Curve

ROC curve graph shows the performance of a classification model. True positive rate (sensitivity) is plotted against the false positive rate (1-specificity) at different classification thresholds. The area under the ROC curve (AUC) gives an index of the performance of the classifier. Higher values of AUC correspond to a good prediction of the model.

In the present study we computed, for each class, the ROC curve graph for each of the 50 network simulations. Then, to obtain a mean ROC curve, we averaged the ROC curve of the 50 simulations as the mean true positive rate for each value of the false positive rate considered on the abscissa.

## Results

**FIGURE 4:**
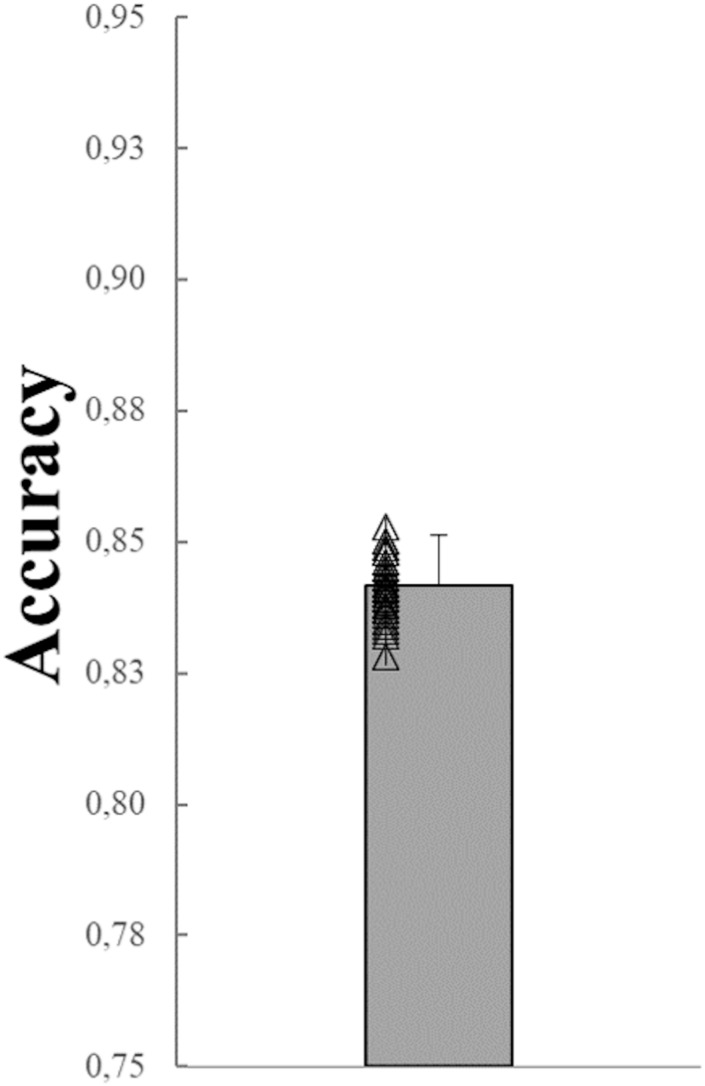
Mean, SD of the mean total accuracy obtained over the 50 network simulations. The mean total accuracy from each of 50 the simulations are superimposed (black empty triangles).

In Figure 4 the mean value (Mean), the corresponding standard deviation (SD) and the distribution of the 50 mean total accuracy values, each corresponding to one of the 50 network simulations (0.839 ± 0.0073) are shown. Values range 0.852–0.820.

**FIGURE 5:**
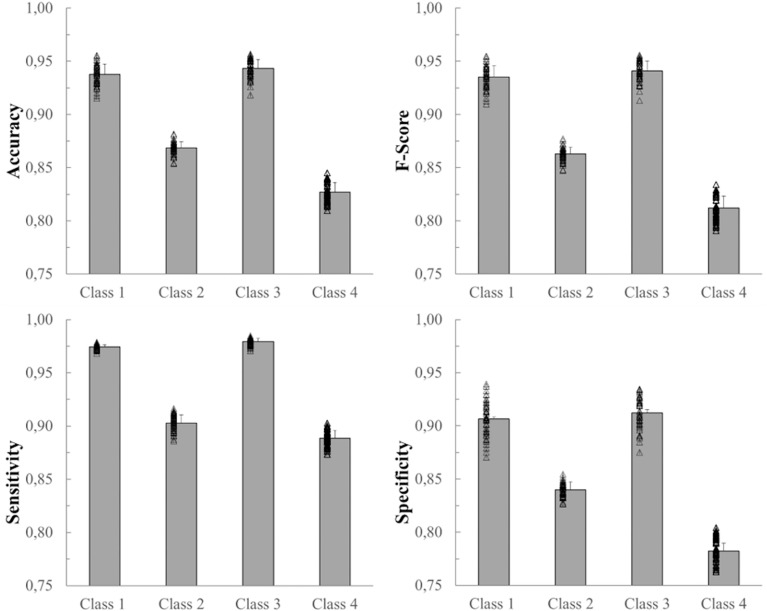
Mean, SD and individual results (black empty triangles) of the 50 network simulations. From top left: accuracy, F-score, sensitivity, and specificity for each of the 4 classes.

Figure 5 shows mean values, SD, and the distributions of the accuracy, F-score, sensitivity, and specificity of each of the four classes. All four variables represented in Figure 5 show a similar trend. Class 3, which corresponds to the lying pose, and Class 1, which corresponds to the standing pose, represent the classes best identified by the net. The network, on the other hand, classified Class 2 (sitting pose) and, especially, Class 4 (“dangerous sitting” pose) with more difficulty for each of the four variables calculated (Figure 5).

**FIGURE 6:**
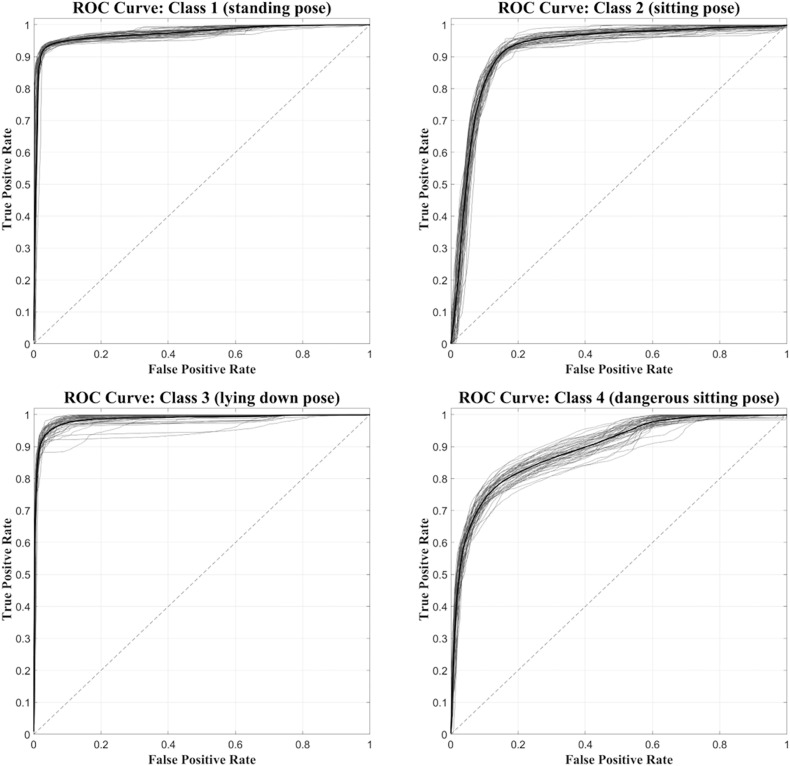
Set of 50 ROC curves calculated for each of the four classes (gray traces). The average ROC curve (black trace) was also calculated and superimposed.

Figure 6 shows, for each class, the ROC curves calculated on the 50 network simulations. The average ROC curve has been calculated for each of the four classes, in order to observe the learning behavior of the network during its 50 simulations. The average ROC curves confirm the observations made previously, i.e., that Class 1 and Class 3 are better identified by the neural network than Class 2 and Class 4. The same results are confirmed also by computing the AUC values for the average ROC curves of the four classes (97.2 for Class1, 92.1 for Class2, 98.5 for Class3 and 89.2 for Class4). Figure 6 also shows the greater variability of the ROC curves relative to Class 4, compared to those obtained with Class 1, Class 2, and Class 3.

**FIGURE 7:**
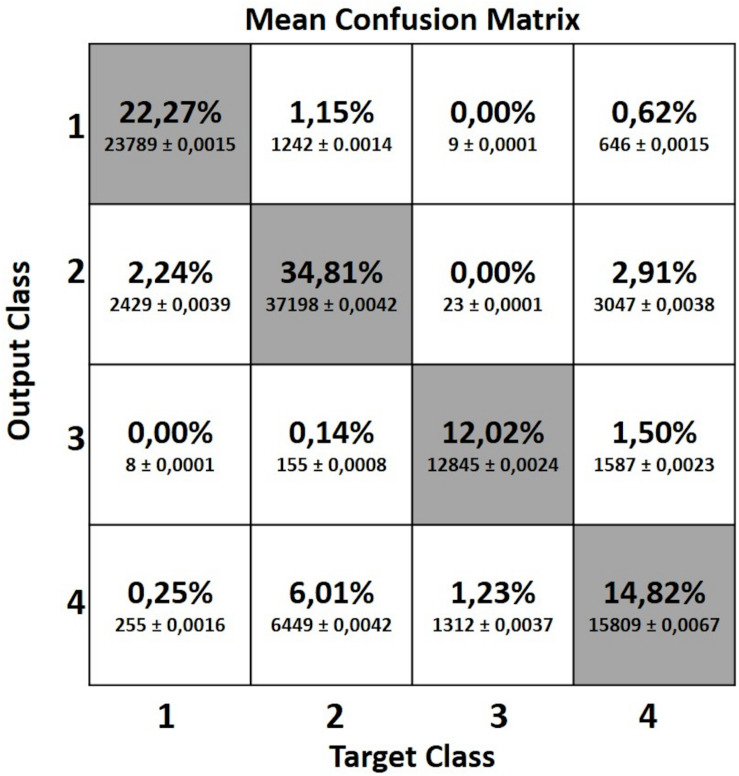
Mean confusion matrix obtained from the 50 network simulations. It represents the False Positive (FP), False Negative (FN), True Negative (TN) and True Positive (TP) values computed for each of the four classes.

Figure 7 shows the mean confusion matrix computed over the entire set of 50 network simulations perform**e** d. It summarizes the average values of the False Positives (FP), False Negatives (FN), True Negatives (TN) and True Positives (TP) for each class.

## Discussion

In order to grant the safety of disabled students living in automated rooms of university dorms while allowing for their independency, their privacy and freedom of movement, we developed a 24/7 monitoring system being able to raise an alarm, either upon request of the student, or automatically when a danger situation is identified. The approach implemented here was based on instrumenting the room with four skeleton-tracking Kinect One devices providing the data for identifying dangerous situations.

In this work we presented a pose recognition system processing the skeleton information provided by the Kinect One devices using a static neural network that classifies the data relative to each frame in one of four classes corresponding to the four poses considered. Three of these (standing, sitting, and lying) represent the most common poses taken by a subject while living in a room, while the fourth (“dangerous sitting”) represents a potential danger situation in which the subject is sitting on a chair with the head forward or backward, that might need an external intervention.

We decided to train and test an MLP model with two hidden layers and a “SoftMax” output layer, in order to classify the four poses described before. After the selection of the attributes and the construction of the database, the MLP neural network was trained and tested 50 times in order to provide data for a statistically reliable description of its performances.

Previous studies have faced similar problems using ML algorithms with good results, although on smaller datasets and asking the subject to maintain the planned poses while facing the camera, i.e., a very favorable condition for the Kinect acquisition, yet unlikely in our project scenario (Patsadu et al., 2012; Visutarrom et al., 2014). Our study considered a less constrained dataset in which 12 subjects were recorded in the defined poses both statically (e.g., lying down) and while moving over the entire room area (e.g., the subjects were walking when assuming the standing pose) for a total of 495728 frames for training and 106802 frames for testing. As a result, our data was more variable in terms of how each subject interpreted the requested poses, and noisier for the different views recorded by each of the four Kinect One devices, which were necessarily frequently sub-optimal.

In spite of these limitations, nonetheless, required to mimic real life conditions, the proposed MLP classifier achieved good results with a total average accuracy of 83.9%. A more detailed inspection of the results relative to the four classes shows that Class 3 and Class 1 are better recognized than the remaining two classes, with average accuracies around 94% (94.3 and 93.8%, respectively). On the other hand, Class 2 and Class 4, both regarding sitting positions yet differing mostly in terms of trunk and head pitch angles, were less accurately recognized (86.9 and 82.7%, respectively), with frames being incorrectly assigned to the two classes (see accuracy values in Figure 5). These lower accuracy values are mainly due to the misclassification errors between Class 2 and Class 4 and vice-versa. Indeed the 6.01% of frames labeled as Class 2 were identified as Class 4 and the 2.91% of Class 4 data were classified as Class 2 (see the mean confusion matrix in Figure 7). At least two plausible reasons can be considered as contributing to this misclassification error in recognizing these two poses. First and foremost, during sitting some articular joints are covered by other body parts, thereby requiring the Kinect One system to reconstruct the positions of the hidden joints and making the resulting data very noisy. Second, despite the careful choice of features as powerful descriptors of body poses while being independent from the physical characteristics of the subjects who participated to the study, the distinction between two kinematically very similar poses is very difficult. The number of features that can help the classifier to distinguish between them is reduced. Only the upper body features may be discriminative and probably, even among these, the normalized vertical positions of the head and cervical vertebrae (Z_1, Z_C7), i.e., the most discriminative joint-related features for the identification of Class 1, 2 and 3, sometimes take comparable values between Class 2 and 4 due to the subjects’ individual interpretation of the description of the “dangerous sitting” pose.

Another relatively important misclassification error was between Class 1 and Class 2 and vice-versa (2.24% Class 1 identified as Class 2 and 1.15% Class 2 identified as Class 1). For the identification of these two poses, the vertical position of the joints (Z_1, Z_C7 and Z_Hc) should be more informative for the MLP network. Nevertheless, in our study this was not so evident probably because some of the data calculated by Kinect One devices are particularly noisy, especially when the subject is not exactly in front of the camera (Rougier et al., 2011; Li et al., 2019). The relative angles and the head and trunk absolute angles do not weight as much in the distinction between the two classes since they assume comparable values. Conversely, lower misclassification error was found for the standing pose (Class 1) and the “dangerous sitting” pose (Class 4) and vice-versa (0.25% Class 1 identified as Class 4 and 0.62% Class 4 identified as Class 1, respectively). In this case, the relative and absolute angles of head and trunk features in the database are more discriminant.

The lowest misclassification error, almost equal to zero, was that between the identification of standing (Class 1) and lying down (Class 3) poses and vice-versa, where the vertical position of the joints is very discriminative.

Considering the assumptions made so far in order to explain the misclassification errors we can hypothesize that an appropriate pre-processing of the data could significantly reduce the number of misclassifications. A classification model requires a reliable and valid dataset to efficiently generate the decision-making rules. To reduce classification errors, the quality of the data provided to the classifier is important during both the training and the usage phases, so that data pre-processing techniques removing anatomically implausible body reconstructions resulting in longer than real limbs or in impossible articular angles may be needed.

## Conclusion

We have proposed an implementation of a pose classification system for monitoring frail individuals in their daily living facilities. Kinect One devices, recording an inhabitant moving in a room in real scenarios, provided skeleton data frames. These data were processed to compute a set of features that make up the database for training and testing a three layers MLP neural network for inhabitant pose recognition (standing, lying, sittingand “dangerous sitting”). We built a database with a large amount of data (over 600,000 frames) in which each pose was described by a set of geometric and vertical joint position features. Despite the data were quite noisy as they were acquired with the subject not necessarily facing the camera, the proposed MLP network achieves a good mean total accuracy of 83.9%.

This work is, to our knowledge, the first attempt to classify human poses based on skeleton tracking data acquired in an ecological daily living scenario, with no constraints on the relative position of the subject with respect to the recording devices, and with an extensive dataset comprising sitting and “dangerous sitting” classes.

This work has been designed for a room tailored to disabled students, but it can be extended to all those categories of individuals living in community environments, such as the elderly, where safety, accessibility and autonomy can be a restriction to participation.

## Data Availability Statement

The raw data supporting the conclusions of this article will be made available by the authors, without undue reservation, to any qualified researcher.

## Ethics Statement

The experimental protocol was conducted in accordance with the Declaration of Helsinki. The participants provided their written informed consent to participate in this study.

## Author Contributions

MS, GB, and BG set up the prototype room for the acquisition session. BG collected the data. GB and BG analyzed and created the database. SR and BG developed and implemented the Machine Learning algorithm. BG wrote the first draft of the manuscript. MS and SR completed and revised the manuscript to reach the final version. All authors contributed to the conception and design of the study and decided on the types of acquisitions to be made, read and approved the submitted version.

## Conflict of Interest

The authors declare that the research was conducted in the absence of any commercial or financial relationships that could be construed as a potential conflict of interest.

## Abbreviations

AAL: Ambient-Assisted Living
ANN: artificial neural network
DL: deep learning
DT: decision tree
HMM: hidden Markov model
IoT: internet of things
KNN: K-nearest neighbor
LR: logistic regression
ML: machine learning
MLP: multi-layer perceptron
NBC: Naïve Bayes classifier
RF: random forest
SVM: support vector machine.
